# Exosomes Derived from Bone Marrow-Mesenchymal Stem Cells Attenuates Cisplatin-Induced Ototoxicity in a Mouse Model

**DOI:** 10.3390/jcm11164743

**Published:** 2022-08-14

**Authors:** Tao Yang, Wei Li, Anquan Peng, Jia Liu, Qin Wang

**Affiliations:** Department of Otolaryngology and Head & Neck Surgery, The Second Xiangya Hospital, Central South University, Changsha 410011, China

**Keywords:** exosomes, hypoxia, bone marrow mesenchymal stem cells, ototoxicity

## Abstract

Background: Both hypoxia preconditioning and exosomes derived from bone marrow mesenchymal stem cells (BMSC-Exo) have been adopted to alleviate hair-loss-related ototoxicity. Whether hypoxic BMSCs-derived exosomes (hypBMSC-Exo) could alleviate cisplatin-induced ototoxicity is investigated in this study. Methods: Cisplatin intraperitoneally injected C57BL/6 mice were trans-tympanically administered BMSC-Exo or hypBMSC-Exo in the left ear. Myosin 7a staining was utilized to detect mature hair cells. Auditory brainstem response (ABR) was assessed to indicate auditory sensitivity at 8, 16, 24, and 32 kHz. The relative expressions of hypoxia-inducible factor-1α (HIF-1α), superoxide dismutase 1 (SOD1), and SOD2 were determined with RT-PCR and Western blot. The content of hydrogen peroxide (H_2_O_2_), malondialdehyde (MDA), SOD, and glutathione (GSH) in the middle turns of the cochlea were measured. Results: Up-regulated HIF-1α expression was observed in hypBMSC-Exo compared with BMSC-Exo. Diminished auditory sensitivity and increased hair cell loss was observed in the cisplatin-exposed mice with increased content of H_2_O_2_ and MDA and decreased content of SOD and GSH, which could be reversed by hypBMSC-Exo or BMSC-Exo administration. It is worth noting that hypBMSC-Exo demonstrated more treatment benefits than BMSC-Exo with up-regulated SOD1 and SOD2 expression in the middle turns of the cochlea tissues. Conclusions: Hypoxic preconditioning may provide a new therapeutic option in regenerative medicine, and hypBMSC-Exo could be utilized to alleviate cisplatin-induced ototoxicity.

## 1. Introduction

Temporary or permanent hearing loss caused by cochleotoxicants may depend on the exposure frequency, intensity, and duration, mainly affecting cochlear hair cells or sensory nerves [1]. In the clinic, cisplatin-induced permanent and bilateral ototoxicity is dose-dependent and progressive [2]; this is a common phenomenon in cisplatin-mediated chemotherapy in solid malignant tumors, ranging from twenty percent to seventy percent [3,4]. Young children are more inclined to experience cisplatin-induced ototoxicity with delayed psychosocial and cognitive development and speech development [5]. Due to the lack of sufficient glutathione (GSH) or the inactivation of the antioxidant system, excessive reactive oxygen species (ROS) induced by cisplatin may contribute to the development and progression of ototoxicity [6]. 

Due to the multilineage differentiation potential and paracrine effects, bone marrow mesenchymal stem cells (BMSCs) have been extensively explored to alleviate ototoxicity in preclinical studies. However, the results are far from satisfactory [7,8]. Multiple preconditioning strategies, such as hypoxia, sound, and hyperthermia, have been adopted to alleviate hair-loss-related ototoxicity [9]. In other words, weak stress performed on the hair cells will protect them against solid stress stimuli. Hypoxia preconditioning has been proposed as an enterprising approach to improve the survival, proliferation, paracrine activity, and exosome production of BMSCs by inducing hypoxia-inducible factor-1α (HIF-1α) to alleviate oxygen tension [10,11,12]. 

Our previous research found that exosomes derived from inner ear stem cells could prevent gentamicin-induced ototoxicity [13], and exosomes derived from cochlear spiral ganglion progenitor cells could attenuate ischemia-reperfusion injury-induced cochlea damage [14]. It is worth noting that exosomes derived from hypoxia-preconditioned adipose stem cells could promote the high-quality healing of the diabetic wound [14], and exosomes derived from hypoxia-preconditioned MSCs could prevent steroid-induced femoral head osteonecrosis [15]. Whether hypoxic BMSCs-derived exosomes (HypBMSC-Exo) could alleviate cisplatin-induced ototoxicity is investigated in this study. 

## 2. Methods and Materials 

### 2.1. BMSCs Isolation and Hypoxia Precondition

C57BL mice (aged 6–8 weeks) were obtained from Cyagen Biosciences Inc., Suzhou, China. The whole animal experimental protocol was approved by the Ethics Committee of Xiangya Hospital. Bone marrow cells were flushed out from femurs, tibias, and iliac bones. After density gradient centrifugation (800 rpm, 5 min), the mononuclear cells were purified with CD11b microbeads (StemCell Technologies, Vancouver, BC, Canada) and seeded into 6-well dishes with a density of 5 × 10^3^ cells/cm^2^ in α-minimum essential medium supplemented with 10% fetal bovine serum (FBS, Gibco, Waltham, MA, USA) (2 mL per well). At the 3rd passage, the cultured BMSCs were evidenced by the positive expression of CD29 and CD105 and the negative expression of CD34 and CD45 confirmed by fluorescence-activated cell sorting (data not shown). Hypoxia precondition (2% O_2_, 5% CO_2_) or normoxia culture (20% O_2_, 5% CO_2_) was carried out using an HF100 Tri-gas incubator (Heal Force, Shanghai, China). 

### 2.2. Exosome Isolation and Determination

BMSCs were cultured in exosome-depleted FBS (Gibco, Grand Island, NY, USA) under hypoxia or normoxia for 48 h. The conditioned media were sequencing-centrifuged (300× *g*, 10 min, 4 °C; 16,500× *g*, 20 min, 4 °C), filtered through 0.22 μm filters, and ultracentrifuged (120,000× *g*, 70 min, 4 °C) to obtain pelleted exosomes, which were resuspended in phosphate-buffered saline (PBS). Exosomes collected from BMSCs under normoxia and hypoxia were denoted as BMSC-Exo and hypBMSC-Exo, respectively.

Nanoparticle tracking analysis was performed to reveal the size distribution of exosomes with NanoSight NS300 equipment (Malvern Instruments Ltd., Malvern, UK). Surface markers of the exosomes (CD63, CD9, and Alix) were detected with Western blots, and the membrane structure was revealed with transmission electron microscopy (TEM) assayed with a JEM-1400Flash Electron Microscope (Jeol, Tokyo, Japan).

### 2.3. Adipogenic and Osteogenic Differentiation of BMSCs

Adipogenic differentiation was performed by cultivating BMSCs in an adipogenic induction solution (10 μg/mL insulin, 1 μM dexamethasone, 0.5 mM isobutylmethylxanthine, 200 μM indomethacin, and 10% FBS in high-glucose Dulbecco’s modified Eagle’s medium (DMEM), Sigma-Aldrich, St. Louis, MO, USA) for 3 days and in an adipose maintenance solution (10 μg/mL insulin, 10% FBS, high-glucose DMEM) for 1 day. All these processes were repeated 3 times, and a total of 14 days were utilized to induce adipogenic differentiation.

High-glucose DMEM containing β-sodium glycerophosphate (10 mM), dexamethasone (1 μM), vitamin C (50 mg/L), and 10% FBS (Sigma-Aldrich) was utilized as the osteogenic differentiation medium. BMSCs were cultured with an osteogenic differentiation medium and renewed at 72 h intervals. The entire process was repeated 3 times, and a total of 14 days were utilized to induce osteogenic differentiation.

Adipogenic- or osteogenic-differentiated BMSCs were fixed with 4% paraformaldehyde for 10 min at room temperature. Alkaline phosphatase (ALP) staining or Oil Red O staining was utilized to detect the osteogenic differentiation or adipogenesis induction, and the images were photographed with a Nikon Eclipse 80*i* microscope (Nikon, Tokyo, Japan).

### 2.4. Cisplatin-Exposed Mice and Exosomes Administration

Ototoxicity was induced through a single i.p. administration of cisplatin (30 mg/kg, 1 mg/mL) in C57BL/6 mice. Half an hour later, BMSC-Exo or hypBMSC-Exo (1 μL, 1.2 µg/µL) was trans-tympanically administered. The animals with the sham surgery were used as the control group.

### 2.5. Auditory Brainstem Response

Auditory brainstem response (ABR) was detected with the TDT System III apparatus (Tucker Davies Technologies, Alachua, FL, USA) to determine the hearing threshold seven days after cisplatin administration. In brief, acoustic stimuli (four frequencies of 8, 16, 24, and 32 kHz; 100 ms duration; intensities ranging from 10 to 100 dB) were directly introduced into the ear canal of anesthetized mice (25 mg/kg xylazine sodium, 100 mg/kg ketamine, i.p.). The subdermal needle electrodes were separately placed in the vertex and the ear pinna to detect the presence of wave V as hearing thresholds. After ABR measurement, cochlear tissues were collected for immunofluorescence staining and the relative mRNA and protein expression analyses.

### 2.6. Quantitative Real-Time RT-PCR (qRT-PCR)

Total RNA was extracted from BMSCs and the middle turns of pooled cochlea tissues with TRIzol reagent (Invitrogen, Waltham, MA, USA) according to the manufacturer’s instructions; the total RNA was then reverse-transcribed into cDNA with an Applied Biosystems High-Capacity cDNA Reverse Transcription Kit. SYBR Green (Roche, Mannheim, Germany) was utilized to detect the amplification with the following procedures: 95 °C for 10 min, 40 cycles of 95 °C for 15 s, and 60 °C for 1 min. Primer sequences were listed: HIF-1α, 5′-TCTCGGCGAAGCAAAGAGTC-3′, reverse primer 5′- AGCCATCTAGGGCTTTCAGATAA-3′; SOD1, forward primer 5′- AACCAGTTGTGTTGTCAGGAC-3′, reverse primer 5′- CCACCATGTTTCTTAGAGTGAGG-3′; SOD2, forward primer 5′- CAGACCTGCCTTACGACTATGG-3′, reverse primer 5′- CTCGGTGGCGTTGAGATTGTT-3′; and GAPDH, forward primer 5′- AATGGATTTGGACGCATTGGT-3′, reverse primer 5′- TTTGCACTGGTACGTGTTGAT-3′. The relative expression data were quantified using the 2^−ΔΔCt^ method after normalizing to GAPDH expression.

### 2.7. Western Blotting

The pooled lysates derived from BMSCs, BMSC-Exo, hypBMSC-Exo, and the middle turns of the cochlea tissues were separated by 12% SDS-PAGE (30 µg protein for each hole) and transferred to PVDF membranes, which were further blocked with 5% non-fat dry milk and incubated with primary antibodies against GAPDH, HIF-1α, SOD1, SOD2, CD63, CD9, and Alix (Santa Cruz, Santa Cruz, CA, USA). Peroxidase-conjugated secondary antibody (Sigma-Aldrich, 1:2000 dilution) was added for 2 h at room temperature, and a Pierce™ Enhanced Chemiluminescence (Thermo Scientific, Waltham, MA, USA) was utilized to obtain the signal. GAPDH was utilized to normalize the relative expression with NIH-Image J1.51p 22. The experiments were repeated 3–4 times with pooled lysates from each group.

### 2.8. Myosin Staining

The middle turns of the cochlea tissues were fixed with 4% paraformaldehyde and permeabilized with 1% Triton X-100 for two hours at room temperature. Permeabilized cochlea tissues were blocked with 5% goat serum, incubated with anti-myosin 7a antibody (1:500 dilution; Proteus Biosciences, 25-6790, Ramona, CA, USA), and further incubated with Texas Red-conjugated goat anti-rabbit secondary antibody (Abcam, ab6719, Cambridge, UK). Images were observed under a Leica SP8 X Confocal Microscope (Leica Microsystems, Biberach, Germany).

### 2.9. Oxidative Stress Assay

Superoxide dismutase (SOD) activity, glutathione (GSH) activity, hydrogen peroxide (H_2_O_2_) level, and malondialdehyde (MDA) content in the cochlea tissues lysates were determined with relevant kits obtained from Nanjing Jiancheng Bioengineering Institute (Nanjing, China). Procedures were performed according to the manufacturer’s instructions using single inner ears. The absorbance was measured with spectrophotometer Evolution™ One UV-Vis Spectrophotometers (Thermo Scientific).

### 2.10. Statistical Analysis

One-way ANOVA followed by Tukey’s multiple comparisons test were utilized to investigate the significance. The significance level was set at a *p*-value < 0.05. All statistical analyses were performed with GraphPad Prism.

## 3. Results

### 3.1. Identification of Primary BMSCs

**Figure 1:**
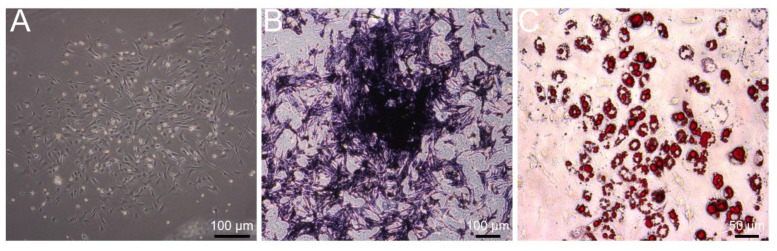
Identification of primary BMSCs from mice. (**A**) Image of BMSCs morphology at the third passage. (**B**) The BMSCs were stained by alkaline phosphatase (ALP) after 14 days of osteogenic differentiation. (**C**) The BMSCs were stained with Oil Red O after 14 days of adipogenesis induction. BMSCs, bone marrow mesenchymal stem cells.

A representative image of BMSCs at passage 3 is shown in Figure 1A, which demonstrated spindle or polygonal morphology with the undifferentiated phenotype. After 14 days of osteogenic differentiation, most BMSCs harbored alkaline phosphatase, demonstrating increased osteogenic activity (Figure 1B). After 14 days of adipogenesis induction, Oil Red O was used to stain neutral triglycerides and lipids to indicate the differentiation of BMSCs into adipocytes (Figure 1C). These data indicated the success of primary BMSCs’ isolation.

### 3.2. Hypoxia-Induced HIF-1α Expressions in BMSCs

**Figure 2:**
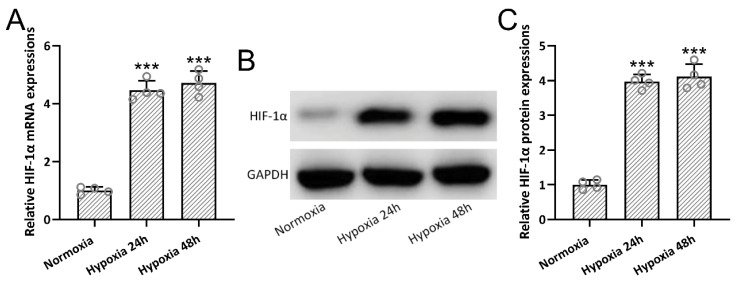
The expressions of HIF-1α in BMSCs after hypoxia treatment. The relative mRNA expressions (**A**) and protein expression (**B**) of HIF-1α at different times after hypoxia treatment. Quantitative Western blot results are demonstrated (**C**). The data are shown with mean ± SD. The experiments were repeated 4 times with pooled cell lysates from each group. *** *p* < 0.001 compared to normoxia. One-way ANOVA followed by Tukey’s multiple comparisons test. BMSCs, bone marrow mesenchymal stem cells; HIF-1α, hypoxia-inducible factor-1α; SD, standard deviation.

Mouse BMSCs were pretreated under hypoxia conditions for 24 h or 48 h. We found that hypoxia preconditioning for 24 h could significantly up-regulate the relative mRNA (Figure 2A) and protein (Figure 2B,C) expression of HIF-1α in BMSCs when compared with normoxia culture. At the same time, prolonged hypoxia preconditioning did not enhance the relative expression of HIF-1α. In the later experiments, 24 h of hypoxia preconditioning was utilized to generate hypBMSC-Exo.

### 3.3. Identification of Hypoxia-Preconditioned BMSC-Derived Exosomes

**Figure 3:**
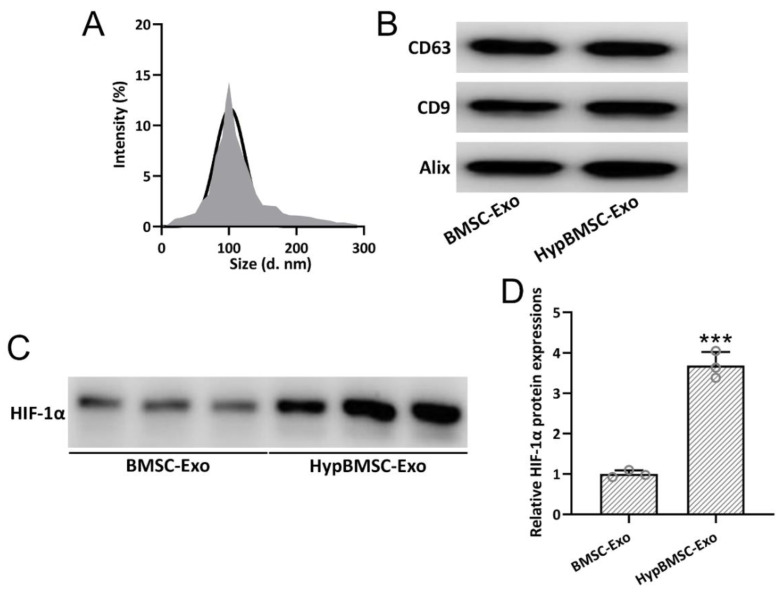
Identification of BMSC-derived exosomes. The size distribution of hypBMSC-Exo and exosomes derived from BMSCs (BMSC-Exo) (**A**). Detection of CD9, CD63, and Alix expression in BMSC-Exo and hypBMSC-Exo by Western blot (**B**). Detection of HIF-1α expression in BMSC-Exo and hypBMSC-Exo by Western blot (**C**,**D**). The data are shown with mean ± SD. The experiments were repeated 3 times with pooled lysates from each group. *** *p* < 0.001 compared to BMSC-Exo. Mann–Whitney test. BMSCs, bone marrow mesenchymal stem cells; HIF-1α, hypoxia-inducible factor-1α; SD, standard deviation.

Exosomes were identified by TEM with single membrane structures, particle size distribution, and exosome-specific protein markers. BMSC-Exo and hypBMSC-Exo showed similar size distribution, with an average size of 110–120 nm (Figure 3A). No differential expression of exosomes markers, such as CD63, CD9, or Alix, was identified in BMSC-Exo or hypBMSC-Exo (Figure 3B). Up-regulated HIF-1α expression was detected in hypBMSC-Exo when compared with BMSC-Exo (Figure 3C,D). These results indicated that hypoxia preconditioning did not change the characterization of exosomes.

### 3.4. HypBMSC-Exo Ameliorated Auditory Sensitivity in Cisplatin-Exposed Mice

**Figure 4:**
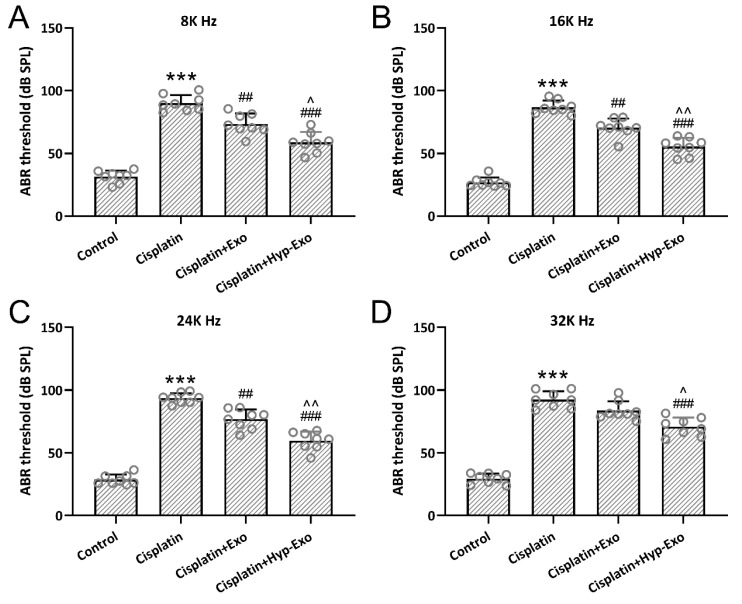
HypBMSC-Exo ameliorated cisplatin-induced hearing loss in vivo. (**A**–**D**) ABR measurements revealed that hypBMSC-Exo reduced hearing thresholds at 8, 16, 24, and 32 kHz, respectively, in cisplatin-exposed mice. The data are shown with mean ± SD. *n* = 8 for each group. *** *p* < 0.001 compared to control, ## *p* < 0.01, ### *p* < 0.001 compared to Cisplatin, ^ *p* < 0.05, ^^ *p* < 0.01 compared to Cisplatin + Exo. One-way ANOVA followed by Tukey’s multiple comparisons test. ABR, auditory brainstem response; hypBMSC-Exo, hypoxic BMSCs-derived exosomes; BMSCs, bone marrow mesenchymal stem cells; SD, standard deviation.

ABR measurement was utilized to indicate the auditory sensitivity. Hearing thresholds were significantly elevated (nearly ∼20 dB) 7 days after cisplatin exposure at 8 kHz (Figure 4A), 16 kHz (Figure 4B), 24 kHz (Figure 4C), and 32 kHz (Figure 4D) in the cisplatin-exposed mice, whereas hypBMSC-Exo or BMSC-Exo treatment could diminish the thresholds significantly. It is worth noting that hypBMSC-Exo could dramatically decrease the hearing thresholds at the four frequencies detected compared with BMSC-Exo. These results indicated that hypBMSC-Exo could restore auditory sensitivity in cisplatin-exposed mice and showed more treatment benefits than BMSC-Exo. 

### 3.5. HypBMSC-Exo Ameliorated Hair Cell Loss in Cisplatin-Exposed Mice

**Figure 5:**
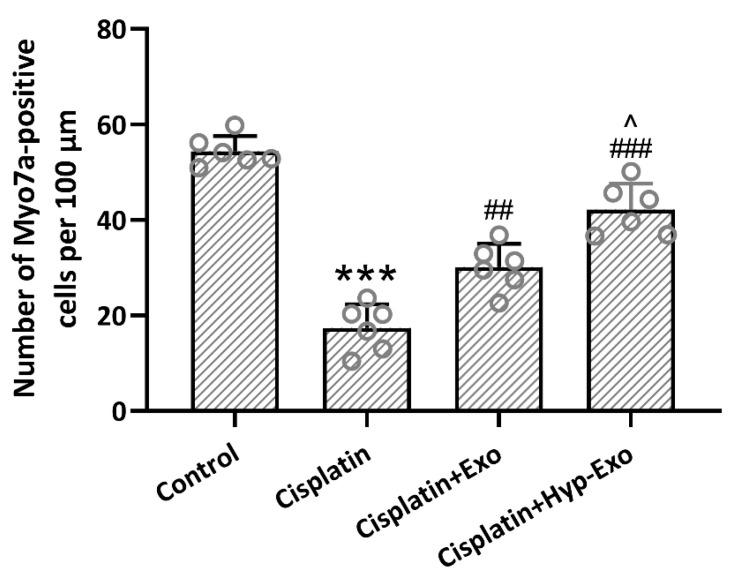
HypBMSC-Exo ameliorated cisplatin-induced hair cell loss in the middle turns of the cochlea. Myosin 7a-positive hair cells after hypBMSC-Exo treatment. The data are shown with mean ± SD. *n* = 6 for each group. *** *p* < 0.001 compared to control, ## *p* < 0.01, ### *p* < 0.001 compared to Cisplatin, ^ *p* < 0.05 compared to Cisplatin + Exo. One-way ANOVA following Tukey’s multiple comparisons test. HypBMSC-Exo, hypoxic BMSCs-derived exosomes.

Myosin 7a staining showed that cisplatin exposure led to a conspicuous ~65% loss of mature hair cells in the middle turn of the cochlea, which could be alleviated by the administration of hypBMSC-Exo or BMSC-Exo (Figure 5). Compared with BMSC-Exo treatment (~45% loss), hypBMSC-Exo (~23% loss) could further alleviate the loss of hair cells. The loss percentage was calculated with data on the number of Myo7a-positive cells per 100 μm. These results demonstrated that HypBMSC-Exo could ameliorate hair cell loss in cisplatin-exposed mice. 

### 3.6. HypBMSC-Exo Activated SOD Antioxidant Signal in Cisplatin-Exposed Mice

**Figure 6:**
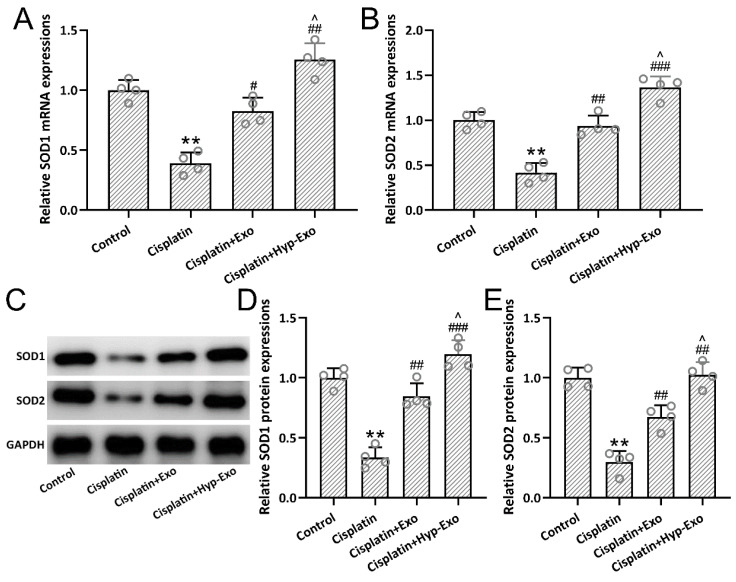
HypBMSC-Exo activated SOD antioxidant signal in cisplatin-exposed mice. The relative mRNA expressions of SOD1 (**A**) and SOD2 (**B**) in the middle turns of the cochlea tissues. The relative protein expressions of SOD1 and SOD2 (**C**) in the middle turns of the cochlea tissues were detected by Western blotting. Quantitative analysis of SOD1 expression (**D**) and SOD2 expression (**E**). The data are shown with mean ± SD. The experiments were repeated 4 times with homogenate from 8 mice in each group. ** *p* < 0.01 compared to control, # *p* < 0.05, ## *p* < 0.01, ### *p* < 0.001 compared to Cisplatin, ^ *p* < 0.05 compared to Cisplatin + Exo. One-way ANOVA followed by Tukey’s multiple comparisons test. BMSCs, bone marrow mesenchymal stem cells; SD, standard deviation; SOD, superoxide dismutase.

Cisplatin-exposed mice demonstrated down-regulated expression of SOD1 (Figure 6A,C,D) and SOD2 (Figure 6B,C,E) in both gene and protein levels, which was restored by the administration of hypBMSC-Exo or BMSC-Exo. Compared with BMSC-Exo treatment, hypBMSC-Exo further up-regulated the relative expression of SOD1 and SOD2 significantly (*p* < 0.05), which indicated the involvement of SOD antioxidant signal in auditory function recovery.

### 3.7. HypBMSC-Exo Ameliorated Cisplatin-Induced Oxidative Stress

**Figure 7:**
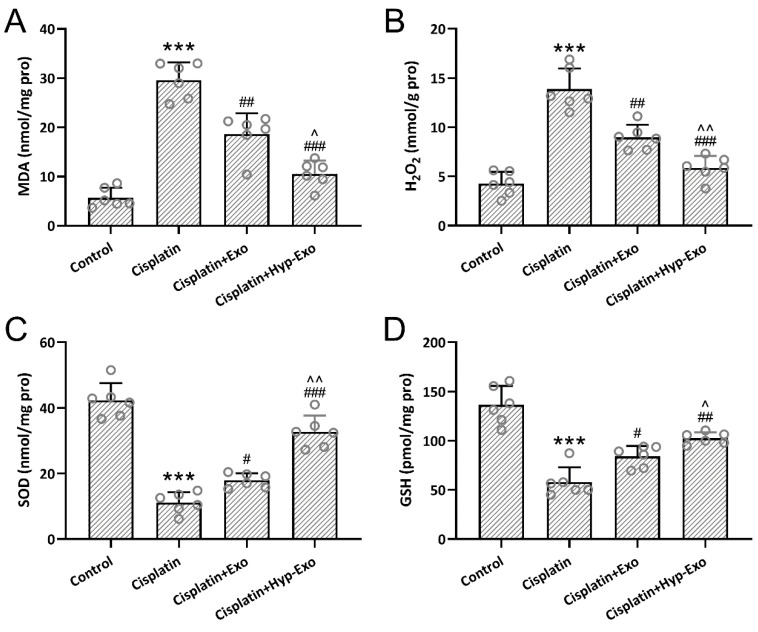
HypBMSC-Exo ameliorated cisplatin-induced oxidative stress in the middle turns of the cochlea. The content of malondialdehyde (MDA) (**A**), hydrogen peroxide (H_2_O_2_) (**B**), superoxide dismutase (SOD) (**C**), and glutathione (GSH) (**D**) in the middle turns of the cochlea. The data were shown with mean ± SD. Eight mice were used for each group. *** *p* < 0.001 compared to control, # *p* < 0.05, ## *p* < 0.01, ### *p* < 0.001 compared to Cisplatin, ^ *p* < 0.05, ^^ *p* < 0.01 compared to Cisplatin + Exo. One-way ANOVA followed Tukey’s multiple comparisons test.

The levels of MDA, H_2_O_2_, SOD, and GSH in the middle turns of the cochlea were measured to indicate oxidative stress. Up-regulated MDA (Figure 7A) and H_2_O_2_ (Figure 7B) and down-regulated SOD (Figure 7C) and GSH (Figure 7D) were observed in cisplatin-exposed cochlea, which was reversed by the administration of hypBMSC-Exo or BMSC-Exo. It was revealed that hypBMSC-Exo treatment further diminished the relative levels of MDA and H_2_O_2_ and increased the relative levels of SOD and GSH significantly compared with BMSC-Exo.

## 4. Discussion

As single-membrane and secreted organelles (30 to 200 nm in diameter), exosomes have the same topology as host cells, and the contents or functions of exosomes are affected by the cellular environment [16,17,18]. Exosomes derived from hypoxia-preconditioned BMSCs demonstrated up-regulated expression of HIF-1α, which could be utilized to alleviate cisplatin-induced ototoxicity with increased auditory sensitivity, alleviated hair cell loss, and diminished oxidative stress when compared with exosomes derived from BMSCs. It is worth noting that the highly disorganized structure of mature hair cells in the middle turn of the cochlea and slight change in ABR threshold shift (~20 dB) were also observed after BMSC-Exo or hypBMSC-Exo treatment, and that the highly disorganized structure indicates the dysregulated auditory function (less ABR threshold shift). These results demonstrated that although no great benefit of ABR threshold shift was obtained, HypBMSC-Exo could ameliorate hair cell loss in cisplatin-exposed mice.

Cisplatin-induced oxidative stress contributes to the cellular damage of the inner ear and ototoxicity [9,19]. In this study, we found that hypBMSC-Exo could be considered a post-exposure treatment option to diminish the production of reactive oxygen species to alleviate hearing loss. Mechanically, diminished MDA and H_2_O_2_ and increased SOD and GSH levels could be attributed to the protective mechanism. As an end product of lipid peroxidation, MDA is formed by the peroxidation of polyunsaturated fatty acids, which can be utilized as a biomarker of lipid oxidative stress [20]. Intraperitoneally given cisplatin could significantly increase MDA levels in serum and cochlear tissue [21]. These findings indicate that cisplatin-induced lipid oxidative stress contributes to the development of ototoxicity. 

The intrinsic antioxidant mechanism, such as the SOD and GSH antioxidant signal, is regulated by hypBMSC-Exo. SOD catalyzes the dismutation of superoxide to H_2_O_2_ and O_2_ [22]. As the most critical low-molecular-weight antioxidant, GSH protects cells from oxidative or xenobiotic damage and maintains redox homeostasis [23,24]. Whether hypBMSC-Exo possesses potential prophylactic roles in antioxidant therapy is an interesting question worth further investigation.

There are some limitations that should be indicated here. Ultrafiltration combined with size-exclusion chromatography will efficiently isolate and separate extracellular vesicles (EV) and EV-free secretomes [25]. More delicate separation methods should be performed in the relevant research to specify the role of exosomes in homeostasis and disease conditions. Hypoxic preconditioning confers significant protection against broadband noise exposure via the activation of HIF-1α in corti [26]. In our analysis, hypoxia preconditioning induced up-regulated HIF-1α expression in BMSC and hypBMSC-Exo; thus, the contribution of HIF-1 in ototoxicity alleviation should be investigated in future studies. 

Ototoxicity induced by cisplatin is an obstacle to the effective treatment of tumors. Our study demonstrated that hypoxic preconditioning may provide new potential therapeutic options for BMSCs in regenerative medicine, and hypBMSC-Exo could be considered as a treatment option in the alleviation of cisplatin-induced ototoxicity.

## 5. Conclusions

Exosomes derived from hypoxic-preconditioned bone marrow mesenchymal stem cells could protect the hair cell loss and inhibit the oxidative stress in cisplatin-induced ototoxicity mice, which may indicate a new research direction for ototoxicity.

## Data Availability

The raw data supporting the conclusions of this article will be made available on request to the corresponding author by email, as requested by our department.
